# Human immune cell engraftment does not alter development of severe acute Rift Valley fever in mice

**DOI:** 10.1371/journal.pone.0201104

**Published:** 2018-07-20

**Authors:** Jessica R. Spengler, Anita K. McElroy, Jessica R. Harmon, JoAnn D. Coleman-McCray, Stephen R. Welch, James G. Keck, Stuart T. Nichol, Christina F. Spiropoulou

**Affiliations:** 1 Viral Special Pathogens Branch, Division of High-Consequence Pathogens and Pathology, Centers for Disease Control and Prevention, Atlanta, GA, United States of America; 2 Division of Pediatric Infectious Diseases, Emory University, Atlanta, GA, United States of America; 3 Divison of Pediatric Infectious Diseases, University of Pittsburgh, Pittsburgh, PA, United States of America; 4 In Vivo Services, The Jackson Laboratory, Sacramento, CA, United States of America; George Mason University, UNITED STATES

## Abstract

Rift Valley fever (RVF) in humans is usually mild, but, in a subset of cases, can progress to severe hepatic and neurological disease. Rodent models of RVF generally develop acute severe clinical disease. Here, we inoculated humanized NSG-SGM3 mice with Rift Valley fever virus (RVFV) to investigate whether the presence of human immune cells in mice would alter the progression of RVFV infection to more closely model human disease. Despite increased human cytokine expression, including responses mirroring those seen in human disease, and decreased hepatic viral RNA levels at terminal euthanasia, both high- and low-dose RVFV inoculation resulted in lethal disease in all mice with comparable time-to-death as unengrafted mice.

## Introduction

Rift Valley fever virus (RVFV; family *Phenuiviridae*, genus *Phlebovirus*) infection causes disease in domestic and wild ruminants, and is often associated with neonatal mortality and abortion. Clinical disease may occur in all age groups but tends to be most severe in young animals. Human infection is typically subclinical or mild; when it occurs, illness generally comprises a flu-like illness with no long-term sequelae. However, human disease can be fatal in 1–3% of cases, or as high as ~50% in patients with hemorrhagic complications. Severe disease may involve acute hepatitis with associated jaundice, renal failure, and hemorrhagic complications. If the patient survives the hepatitis, neurological manifestations may include signs of encephalitis and vision loss [1].

Animal models of Rift Valley fever (RVF) include mice, rats, hamsters, and non-human primates; livestock can be used to experimentally investigate natural disease [2]. In mice, RVFV infection results in rapid and uniformly lethal disease due, in large part, to virally mediated innate immune suppression [3,4]. In comparison, stronger immune control of RVFV in humans is thought to be critical in reducing disease severity. However, the specific human immune contributors to disease modulation are not known. Here we used SGM3 mice reconstituted with human immune cell populations via stem cell engraftment (Hu-NSG-SGM3) to investigate whether the presence of these cells in mice, and the resultant human-like immune response, could better regulate viral replication to confer a less severe clinical presentation, as seen in human disease.

## Materials and methods

### Ethics statement

Work with infectious virus was performed in the biosafety level 3 facilities of the Centers for Disease Control and Prevention (CDC). Sample inactivation and removal were performed according to standard operation protocols approved by the local Institutional Biosafety Committee and the Laboratory Safety Review Board. All animal experiments were approved by the CDC Institutional Animal Care and Use Committee (IACUC; #2736SPEMOUC) and performed in compliance with the guidelines of IACUC and of the Association for Assessment and Accreditation of Laboratory Animal Care (AAALAC) by certified staff in an AAALAC-approved facility.

### Mice

Ten female NSGS mice (NSG-SGM3, stock no. 013062) and fourteen female humanized SGM3 mice, irradiated and injected via tail vein at 4 weeks of age with human umbilical cord blood-derived CD34^+^ hematopoietic stem cells from 1 of 4 donors (Hu-NSG-SGM3, stock no. 701362), were obtained from Jackson Laboratories. Engraftment levels were assessed once in all humanized mice 12 weeks (wk) post engraftment. Mice were housed in a climate-controlled laboratory with a 12 h day/12 h night cycle; given sterile water, bedding, and food; and housed 1 to 5 mice/cage in an isolator caging system. Mice were humanely euthanized with isoflurane vapors when clinical illness scores based on weight loss (>20%), piloerection, neurological signs, ataxia, dehydration, or dyspnea indicated that the animal was in distress or in the terminal stages of disease.

### Virus inoculation

Humanized mice were equally distributed among experimental groups based on age and subsequently on engraftment level. Background unengrafted NSGS and Hu-NSG-SGM3 mice were inoculated intramuscularly bilaterally in the caudal thighs with target dose of 10^4^ TCID_50_ (Hi) or 10^1^ TCID_50_ (Lo) of RVFV (rZH-501 [Genbank accession no. DQ380149.1, DQ380200.1, DQ375406.1] sequence confirmed, 2^nd^ passage after rescue) diluted in Dulbecco’s modified Eagle’s medium (DMEM). Delivered Hi and Lo doses were determined to be 1.29 × 10^4^ and 1.29 × 10^1^ TCID_50_, respectively, by back-titrating Hi dose inoculum. Ratio of PFU to TCID_50_ in this virus stock was 0.57 ± 0.08. Hi and Lo inoculation groups of humanized mice each included 7 animals; 4 were 13 wk post engraftment and 3 were 19 wk post engraftment at the time of inoculation. NSGS Hi and Lo inoculation groups had 5 10-wk-old mice each.

### Quantitative RT-PCR

RNA was extracted from blood and homogenized tissue samples using the MagMAX-96 Total RNA Isolation Kit (Thermo Fisher Scientific) on a 96-well ABI MagMAX extraction platform with a DNase I treatment step, according to manufacturer’s instructions (protocol AM1830 DW). RNA was quantitated using a one-step real-time RT-PCR targeting the RVFV L gene sequence [5], with values normalized to 18S transcript levels (SuperScript III Platinum One-Step qRT-PCR Kit, Thermo Fisher Scientific). Relative viral L genome copy numbers were calculated using standards prepared from in vitro-transcribed L genomic RNA and are expressed per μL of eluted RNA.

### Cytokine expression

Plasma samples were gamma-irradiated (5.0 × 10^6^ rads), then analyzed using a human cytokine magnetic 25-plex panel (50 μL of sample; Thermo Fisher Scientific, LCH0009M) and a mouse cytokine magnetic 26-plex ProcartaPlex Panel 1 (25 μL of sample; Thermo Fisher Scientific, EPXR260-26088-901) per manufacturer’s instructions using a 2 h incubation. Samples were read on a Luminex 200 platform.

### Statistical analyses

Survival was analyzed by log-rank (Mantel-Cox) test. Viral RNA levels were analyzed to determine overall significance between sample types (one-way ANOVA) and within sample types (two-way ANOVA). Two-way ANOVA was also used to analyze group differences in human and mouse cytokine expression. All ANOVA analyses were conducted with Tukey’s multiple comparison tests. All analyses were performed using GraphPad Prism v7.0 software.

## Results

### Presence of human immune cells does not prolong clinical course or reduce severity of RVFV infection in mice

To investigate the clinical course and pathogenesis of RVFV in humanized mice, Hu-NSG-SGM3 mice were inoculated with either 10^4^ TCID_50_ (Hi) or 10^1^ TCID_50_ (Lo) of recombinant RVFV strain ZH-501. As mice are very sensitive to RVFV infection, and direct routes (e.g, intraperitoneal inoculation) often result in a more acute disease course [6,7], the mice were inoculated intramuscularly in an effort to slow disease progression. Engraftment levels were assessed in all humanized mice prior to inoculation at 12 wk post engraftment (Table 1). As human cell populations can vary with time post-engraftment and may affect outcome, mice either 13 or 19 wk post engraftment were included within the Hi and Lo inoculation groups. Two groups of five NSGS mice, the background strain of the humanized mice, were also inoculated with an equivalent Hi or Lo RVFV dose.

**Table 1:** Human immune cell reconstitution in mouse blood. Human cell engraftment assessed by flow cytometry 12 weeks post engraftment in all mice.

	Mouse (Donor ID)	Wk PE at day 0	RVFV dose (TCID_50_)	hCD45^+^ total %	hCD19^+^ (B cells) % of hCD45^+^	hCD3^+^ (T cells) % of hCD45^+^	hCD33^+^ (myeloid) % of hCD45^+^	hCD45^-^ total %
**Hi**	5083–07	13	10,000	49.8	76.2	0.3	20.2	50.2
	5083–08	13	10,000	55.7	80.1	0.2	16.9	44.3
	5083–38	13	10,000	58.2	78.7	0.5	18.1	41.8
	5082–17	13	10,000	69.5	83.4	2.6	10.1	30.5
	2162–02	19	10,000	33.2	13.6	75.0	10.5	66.8
	2162–04	19	10,000	27.4	84.6	0.8	11.4	72.6
	2162–05	19	10,000	32.9	75.2	7.2	13.4	67.1
**Lo**	5082–15	13	10	74.3	84.5	1.3	10.1	25.7
	5082–29	13	10	73.5	77.5	1.0	16.7	26.5
	5083–17	13	10	49.3	67.6	3.1	25.2	50.7
	5084–20	13	10	40.0	81.3	1.0	11.9	60.0
	2162–01	19	10	26.1	45.7	33.7	16.1	73.9
	2162–03	19	10	41.6	41.1	42.0	12.4	58.4
	2162–07	19	10	31.8	65.5	23.4	8.5	68.2

PE, post-engraftment; day 0, day of virus inoculation. Hi and Lo refer to RVFV dosing.

**Fig 1:**
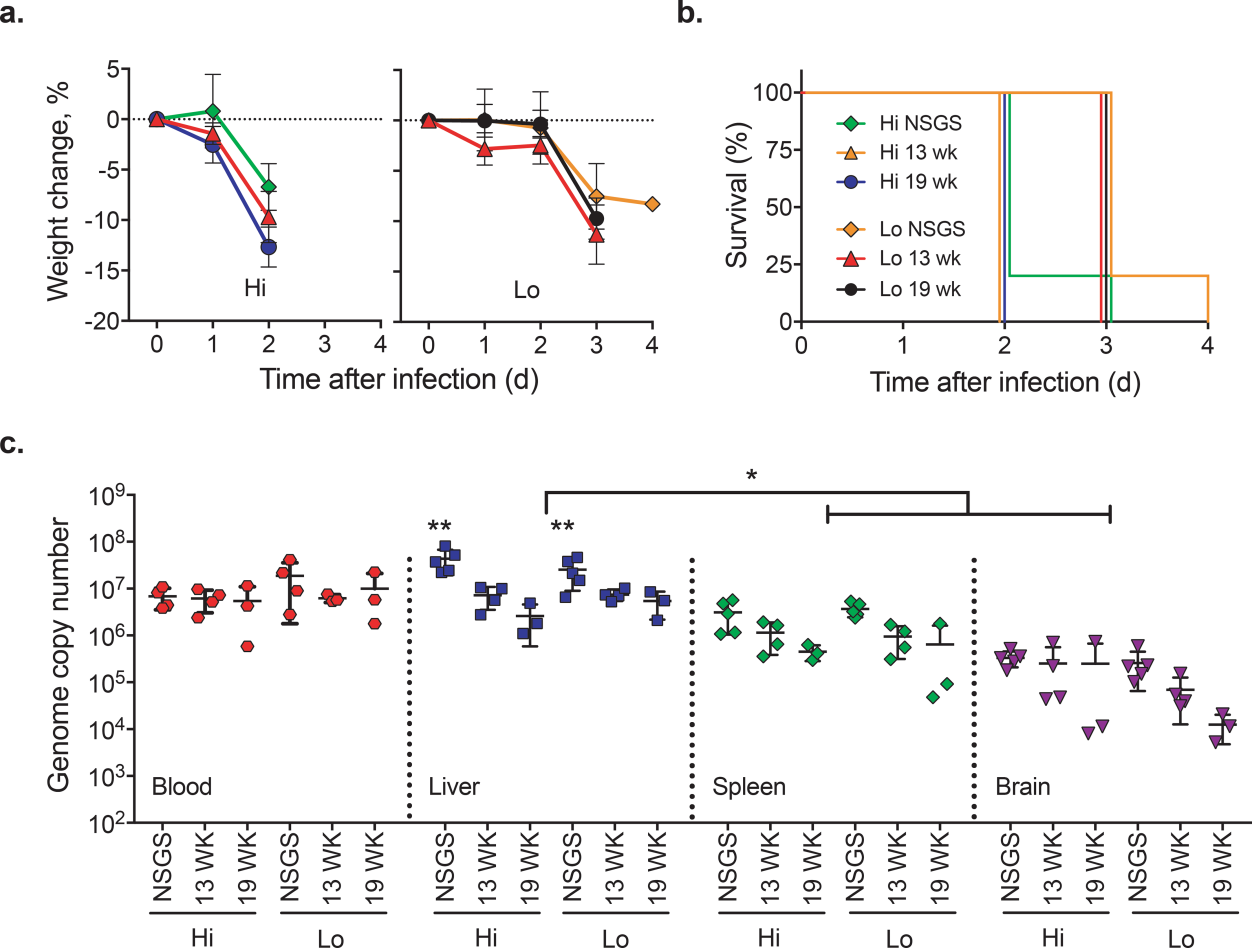
Weight change, survival, and viral RNA levels in RVFV-inoculated humanized mice. (A) Weight change (mean ± SD) and (B) survival in unengrafted NSG-SGM3 (NSGS) mice and Hu-NSG-SGM3 mice inoculated intramuscularly with 10^4^ TCID_50_ (Hi) or 10^1^ TCID_50_ (Lo) of RVFV. Humanized mice were inoculated either 13 or 19 weeks (wk) post engraftment, as indicated. (C) Viral RNA genome copy number (per μL of RNA) normalized to 18S in blood, liver, spleen, and brain collected at time of terminal euthanasia (NSGS mice: 2–3 DPI for Hi or 3–4 DPI for Lo; Hu-NSG-SGM3 mice: 2 DPI for Hi or 3 DPI for Lo) determined by qRT-PCR (mean ± SD). Blood was obtained from all but 3 mice (Lo-13-wk-1 [ID 5082–15], and one mouse each in the Hi- and Lo-NSGS groups). Significant at confidence of *p ≦ 0.05 (NSGS vs. Hu-NSG-SGM3 at same dose); **p < 0.0001 (mean in liver of all groups vs. in spleen or brain).

Humanized mice inoculated with Hi-dose RVFV all succumbed to disease at 2 days post infection (DPI), and with Lo-dose RVFV at 3 DPI (significant at p = 0.0253; Fig 1). Disease onset was rapid in all animals; notable weight loss from previous day (~10%) was only observed in the 24 h period prior to meeting euthanasia criteria. With the exception of one mouse found dead at 3 DPI (Lo-13-wk-1 [ID 5082–15]), all humanized mice were humanely euthanasia at end-point criteria based on clinical signs including severe neurological signs (tremors, seizure) and/or moribundity. All unengrafted NSGS mice were humanely euthanized (Hi at 2–3 DPI; Lo at 3–4 DPI) due to comparable clinical signs with the exception of one animal in each group that succumbed prior to euthanasia.

Level of human immune cell engraftment 12 wk post engraftment (Table 1) or age post engraftment did not affect outcome. Clinical disease was comparable, and survival was not significantly different between humanized mice and background NSGS mice in the Hi or the Lo dose groups. Viral RNA was detected in all blood and tissue samples, including liver, spleen, and brain. Although viral RNA levels were high in all groups in the liver (≧1.10 × 10^6^ copies), levels were significantly higher in both Hi- and Lo-NSGS mice than in corresponding Hi- and Lo-Hu-NSG-SGM3 mice at either age post engraftment (p <0.0001); no significant differences in viral RNA levels between experimental groups were detected in the blood, spleen, or brain. Overall, the mean viral RNA copy number of all liver samples was significantly higher than in spleen (p = 0.05) or brain tissue (p = 0.03)

Engraftment levels are dynamic, but baseline levels determined 12 wk post engraftment can indicate variation in overall engraftment success (Table 1). B cell (hCD19^+^) levels 12 wk post engraftment were comparable between the 13-wk and 19-wk post engraftment groups and not associated with time to death. T cell (hCD3^+^) levels 12 wk post engraftment were much lower in the 13-wk post engraftment group (mean 1.3%, range 0.2–3.1%) than in the 19-wk group (mean 30.4%, range 0.8–75.0%). However, these variations did not alter outcome within each infection group. Mean myeloid cell (hCD33^+^) levels were slightly higher in the 13-wk group (16.2%) than in the 19-wk group (12.0%), but similarly did not result in phenotypic differences.

### RVFV infection in humanized mice increases expression of both human and mouse cytokines

**Fig 2:**
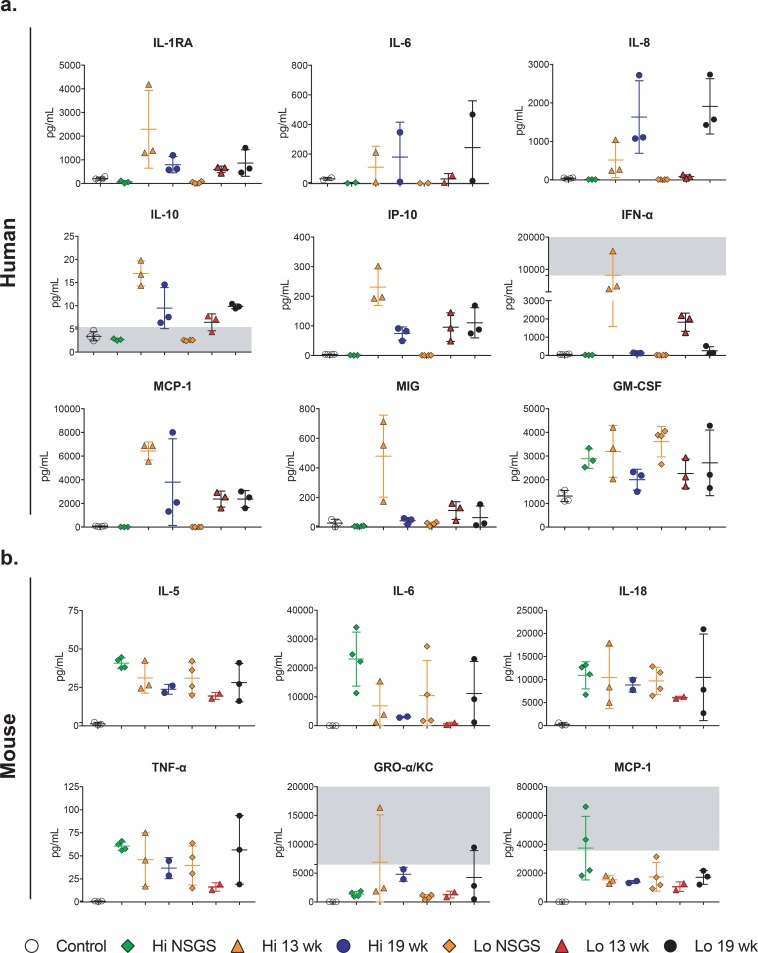
Human and mouse cytokine expression in RVFV-inoculated humanized mice. Background control NSGS mice or Hu-NSG-SGM3 mice at 13 or 19 wk post engraftment were inoculated intramuscularly with 10^4^ TCID_50_ (Hi) or 10^1^ TCID_50_ (Lo) of RVFV; samples were collected at terminal euthanasia (3 DPI for Lo; 2 DPI for Hi). Human (A) and mouse (B) cytokine expression in plasma of Hu-NSG-SGM3 determined by multiplex bead-based assays (mean ± SD). Illustrated is a subset of analytes that, in general, demonstrated the most pronounced increases in expression; complete human 25-plex and mouse 26-plex array data are available in S1 Table and S2 Table, respectively. In addition to samples not obtained due to acute terminal disease, samples excluded due to insufficient volume from human cytokine analysis include Hi-13-wk-2 (ID 5083–08) and Hi-NSGS-2, and from mouse cytokine analysis Lo-13-wk-4 (5084–20), and Hi-13-wk-2 (ID 5083–08). Historical samples from mock-inoculated (control, open circles) Hu-NSG-SGM3 mice were used to determine baseline expression in both panels. Shading indicates areas outside of the dynamic range of the assay, as determined by the standard curve run in conjunction with samples.

Human cytokine production was detected in plasma of RVFV-inoculated humanized mice using a 25-plex Luminex assay (Fig 2A, S1 Table). As expected, all human cytokines were below the limit of detection in unengrafted NSGS with the exception of human GM-CSF that they express transgenically and IFN-α, which may represent some low level of cross-reactivity. Human cytokine expression levels in terminal Hu-NSG-SGM3 exhibited high variability. Analyte expression was not uniform across experimental groups, and the groups with the highest expression of any particular analyte varied (i.e., if one group expressed one analyte at highest levels, it did not necessarily express the highest levels of another analyte). Human analytes in which significant differences were observed between 2 or more experimental groups included IL-1RA, IL-2R, IL-8, IFN-α, MCP-1, and GM-CSF. However, IL-2R was increased in all humanized mice compared to unengrafted NSGS, irrespective of infection status. In addition, IP-10, IL-6, and MIG are graphically depicted due to involvement in human disease.

Humanized mice have circulating human immune cells, but the majority of tissue cells are of murine origin. The relative contribution of mouse responses in this model is currently unknown. Thus, we performed additional analyses to detect and quantitate mouse cytokine production in the plasma of RVFV-inoculated humanized mice using a 26-plex Luminex assay (Fig 2B, S2 Table). Plasma collected at terminal timepoints from unengrafted NSGS mice inoculated with Hi- or Lo-dose RVFV was used in this assay to provide a relative measure of expression levels in non-humanized mice that succumbed to RVFV infection (S2 Table). Murine analytes in which significant differences were observed between 2 or more experimental groups included IL-6, IL-18, and MCP-1. However, while not at significant levels, increases compared to uninfected Hu-NSG-SGM3 controls were also observed in IL-5, TNF-α, and mGRO-α/KC in infected mice. Differences in mouse cytokine expression between Hu-NSG-SGM3 and unengrafted NSGS were only observed in IL-6 (decreased in Hi-13-wk and Hi-19-wk vs. Hi-NSGS, p < 0.0001; decreased in Lo-13-wk vs. Lo-NSGS, p = 0.005) and MCP-1 (decreased in Hi-13-wk and Hi-19-wk vs. Hi-NSGS, p < 0.0001; Lo-13-wk and Lo-19-wk vs. Lo-NSGS, not significant).

## Discussion

Humanized mice—mice that express human genes or contain human cells and/or tissues (e.g., transgenic mice or xenograft mouse models)—have shown remarkable promise in modeling human disease [8]. Notably, for agents such as viruses that cause hemorrhagic fever in humans but frequently do not cause disease in animals without severe immunodeficiency or serial virus adaptation, humanized mice offer new opportunities for investigating disease pathogenesis and assessing investigational therapeutics. Here, we inoculated Hu-NSG-SGM3 mice, previously characterized in other viral hemorrhagic fever infections [9,10], with RVFV in an attempt to more faithfully recapitulate human disease. However, despite human CD45^+^ cell engraftment and detection of both mouse and human cytokine responses, RVFV infection in humanized mice resulted in acute, uniformly lethal disease by 2–3 DPI.

In rodents, acute lethal disease is characterized by severe hepatic damage, although virus is detected in other visceral and central nervous system tissues [11]. Some mouse strains, such as Balb/c, may exhibit a more prolonged clinical course that includes pathological changes in the brain [11]. In this study, clinical presentation of RVFV infection in humanized mice was comparable to that seen in other rodent models infected with wild-type RVFV, with acute onset and rapid progression to severe or lethal disease. This rapidly lethal phenotype is due to the fact that RVFV is extremely efficient at antagonizing the innate immune response in mice via the action of the viral interferon antagonist NSs [12]. Use of attenuated virus strains to slow disease progression or alter disease phenotype has been one approach to model human disease. Mice inoculated with RVFV containing deletions of the NSs protein have been used to investigate RVF disease, including the encephalitic form [13]. In this model, the encephalitic form of disease occurs following a prolonged clinical course, which can extend weeks post infection, suggesting a role for the adaptive immune response in preventing encephalitis. In support of this hypothesis, in the attenuated mouse model, B cells and CD4^+^ T cells, but not CD8^+^ T cells, were critical for mediating viral clearance, even in the presence of a functional innate response [13]. However, use of this attenuated virus model is limited, since it requires altering host adaptive responses to manifest the encephalitic phenotype.

In human RVF disease, serum virus concentrations and disease severity are positively correlated [14], but the role of the immune system in protection from RVFV disease is not as well understood. Increased levels of cytokines IL-8, IP-10, IL-10, MCP-1, and MIG have been associated with a fatal outcome [15]. In a contrasting study, 2 pro-inflammatory cytokines, sCD40L and GRO, were associated with survival during natural infection [16]. Both studies demonstrated an association between IL-10 and fatal outcome, indicating that cytokine dysregulation is a feature of severe human disease. Human IL-1RA, IL-8, IL-10, IP-10, MCP-1, and MIG, which were upregulated in a subset or all of our RVFV-infected humanized mice, are also upregulated in patients who succumb to infection. However, levels of other analytes measured in our study did not mirror those in human infection. For example, increased IFN-α levels observed in the 13-wk post engraftment group may be due to higher myeloid engraftment and did not alter outcome. Increased IL-6 and IL-12 expression has been reported in fatal RVF cases, although not at levels of statistical significance. Increased IL-6 expression was observed in all of the humanized mouse infection groups except Lo-13-wk, but IL-12 expression in these mice was unimpressive, perhaps due to low T cell engraftment. Most IL-6 expression is from non-hematopoietic cells [17], but we only measured hematopoietic cell production, limiting our ability to dependably compare expression to human disease.

Small animal models offer many logistical advantages for investigating immune responses elicited by RVFV infection in humans. However, non-human primates continue to be the model that most closely mimics human illness; in certain species, disease can be mild, and progression to severe disease is observed in only a subset of infected animals [2]. While RVFV lethality in Hu-NSG-SGM3 mice was similar to that previously described in other rodent models, results may vary between humanized mouse models, partly because these models can be highly variable in derivation and experimental outcome. Although described almost 2 decades ago, these animals are still nascent in terms of characterization and potential for future advancement. New models are being developed and characterized in increasing numbers. The details of each model, including engraftment levels, age post-engraftment, and donor representation should all be considered prior to future experimental attempts.

Notably, our data emphasize the importance of murine cell populations in humanized mouse studies and highlight the value of assessing murine responses in data interpretation and future humanized mouse model development. Likely due to highly permissive host cells and robust viral antagonism of the interferon response, both murine and human responses in this model were insufficient to overcome RVFV replication and dissemination. While humanized mice remain an innovative and promising option in viral hemorrhagic fever research, our data demonstrate that the presence of human immune cells alone does not always confer a more human-like outcome, and that these cells cannot universally overcome murine responses to certain agents.

## Supporting information

S1 Table Human cytokine expression in plasma of mock- and RVFV-infected humanized mice. Human cytokine levels in plasma of mock- and RVFV-inoculated humanized mice at terminal timepoints. Historical samples from mock-inoculated (control) SGM3 humanized mice were used to determine baseline expression. Values are expressed as the mean (range) in pg/mL.
(DOCX)

S2 Table Mouse cytokine expression in plasma of mock- and RVFV-infected humanized mice. Mouse cytokine levels in plasma of mock- and RVFV-inoculated humanized mice at terminal timepoints. Historical samples from mock-inoculated (control) SGM3 humanized mice were used to determine baseline expression. Values are expressed as the mean (range) in pg/mL.
(DOCX)
