# Supplementary material for: Human immune cell engraftment does not alter development of severe acute Rift Valley fever in mice
Source: PLoS One. 2018 Jul 20;13(7):e0201104. doi: 10.1371/journal.pone.0201104 (PMC6054394; doi:10.1371/journal.pone.0201104)
Supplement: S1 Table — Human cytokine levels in plasma of mock- and RVFV-inoculated humanized mice at terminal timepoints. Historical samples from mock-inoculated (control) SGM3 humanized mice were used to determine baseline expression. Values are expressed as the mean (range) in pg/mL. (DOCX) [file pone.0201104.s001.docx]

**S1 Table. Human cytokine expression in plasma of mock- and RVFV-infected humanized mice.** Human cytokine levels in plasma of mock- and RVFV-inoculated humanized mice at terminal timepoints. Historical samples from mock-inoculated (control) SGM3 humanized mice were used to determine baseline expression. Values are expressed as the mean (range) in pg/mL.

|  |  |  | **RVFV-inoculated** | | | | | |
| --- | --- | --- | --- | --- | --- | --- | --- | --- |
| **Cytokine** | **LLDR (pg/mL)** | **Control** | **Hi-NSGS** | **Hi 13-wk** | **Hi 19-wk** | **Lo-NSGS** | **Lo 13-wk** | **Lo 19-wk** |
| IL-1β | 9.88 | 25.9 (0.99–47.5)* | 7.7 (1.7–17.7)* | 11.0 (4.08–20.9) | 12.4 (8.29–15.7)* | 3.4 (0.6–8.7)* | 5.6 (5.6, 5.6)* | 19.9 (12.0–26.8) |
| IL-1RA | 145.87 | 204.5 (135.2–292.4)* | 63.6 ( 13.3–113.9)* | 2290.5 (1294.8–4187.0) | 794.2 (579.0–1189.9) | 35.4 (3.4–89.4)* | 596.2 (435.4–686.8) | 865.3 (459.9–1504.2) |
| IL-2 | 13.27 | 10.3 (4.7–16.3)* | 2.6 (1.2–5.1)* | 5.7 (3.4–9.3)* | 5.7 (3.7–8.1)* | 1.7 (0.8–3.4)* | 3.1 (1.3–4.3)* | 6.7 (3.0–9.9)* |
| IL-2R | 28.76 | 1435.4 (899.4–1702.6) | 25.9 (18.2–32.7)* | 1454.4 (1122.1–1915.4) | 1334.2 (1239.5–1447.0) | 19.5 (15.6–23.2)* | 1055.1 (943.3–1185.7) | 1577.3 (1300.0–1977.8) |
| IL-4 | 34.51 | 12.7 (6.6–18.5)* | 17.1 (12.7–22.4)* | 24.8 (17.9–38.8)* | 18.1 (13.8–20.6)* | 14.6 (8.7–17.3)* | 14.1 (7.9–21.3)* | 18.5 (16.5–22.0)* |
| IL-5 | 11.70 | 11.1 (8.7–15.4)* | 2.6 (2.4–2.8)* | 18.5 (13.2–26.3) | 21.9 (14.7–29.1) | 2.5 (2.3–2.7)* | 12.2 (10.2–14.7)* | 23.9 (20.5–27.7) |
| IL-6 | 7.87 | 39.5 (14.2–61.9) | 3.3 (2.9–3.8)* | 211.5 (112.7–325.0) | 346.6 (87.9–798.7) | 2.7 (2.5–3.0)* | 57.5 (35.6–97.1) | 467.4 (240.3–676.9) |
| IL-7 | 13.68 | 112.2 (52.9–190.2)* | 19.6 (4.8–35.4)* | 129.7 (88.3–181.5) | 145.9 (124.8–166.9)* | 19.1 (8.7–24.4)* | 49.7 (33.0–65.9) | 103.5 (90.6–112.1) |
| IL-8 | 12.24 | 43.1 (22.3–56.3)* | BLD | 518.8 (242.2–1044.4) | 1633.6 (1075.0–2720.3) | BLD | 88.1 (44.1–134.8) | 1911.9 (1428.6–2736.2) |
| IL-10 | 5.40 | 3.4 (2.4–4.8)* | 2.7 (2.5–2.9)* | 17.0 (14.4–19.8) | 9.5 (6.36–14.6) | 2.5 (2.4–2.6)* | 6.4 (4.35–7.77)* | 9.9 (9.40–10.4) |
| IL-12 | 10.31 | 49.3 (25.1–70.6) | 7.1 (4.8–8.7)* | 72.8 (43.1–95.6) | 50.8 (30.5–62.3) | 7.3 (4.5–8.4)* | 32.4 (29.6–34.1) | 44.9 (40.4–47.7) |
| IL-13 | 16.50 | 13.7 (10.5–20.4)* | 9.6 (7.9–10.8)* | 16.2 (12.4–20.4)* | 13.9 (10.9–17.2)* | 9.2 (7.9–10.3)* | 10.1 (9.3–11.6)* | 16.4 (14.3–17.8)* |
| IL-15 | 66.75 | 338.4 (101.9–661.4) | 75.0 (57.6–96.8)* | 251.4 (157.4–415.5) | 266.7 (220.2–332.2) | 55.1 (49.3–65.8)* | 103.8 (11.8–206.3)* | 362.6 (200.8–484.8) |
| IL-17A | 26.89 | 49.7* | 2.1 (1.6–2.7)* | 7.7 (3.6–15.4)* | 2.5 (1.6–3.6)* | 1.8 (1.2–1.9)* | 1.0 (0.3–1.6)* | 4.2 (3.6–4.8)* |
| IP-10 | 6.38 | 3.7 (3.3–4.1)* | 0.9 (0.5–1.4)* | 231.0 (193.2–302.4) | 74.1 (49.1–91.2) | 0.6 (0.4–0.7)* | 95.7 (48.7–145.3) | 110.4 (74.6–168.8) |
| IFN-α | 11.23 | 61.4 (48.6–85.6) | 24.0 (19.0–28.0) | 8161.5 (3917.1–15737.0)* | 129.5 (111.1–149.1) | 20.6 (15.5–24.2) | 1821.0 (1254.8– 2186.5) | 260.0 (119.9–518.5) |
| IFN-ɣ | 7.12 | 17.3 (1.2–48.0)* | 2.5 (2.2–2.9)* | 13.9 (9.94–15.1) | 6.8 (5.0–8.4)* | 2.2 (2.1–2.6)* | 7.3 (3.5–12.2)* | 8.5 (4.7–14.0)* |
| TNF-α | 8.57 | 3.0 (1.1–4.5)* | 1.5 (1.4–1.6)* | 9.1 (5.20–11.8)* | 7.0 (5.4–9.5)* | 1.5 (1.4–1.8)* | 3.4 (3.0–4.1)* | 13.6 (7.78–21.1)* |
| Eotaxin | 4.32 | 0.9 (0.7–1.1)* | 0.4 (0.4–0.5)* | 2.0 (1.1–2.9)* | 1.0 (0.9–1.0)* | 0.4 (all at 0.4)* | 1.1 (0.9–1.3)* | 1.1 (1.1–1.2)* |
| MCP-1 | 22.53 | 79.4 (46.1–98.7) | 13.0 (5.7–17.8)* | 6430.0 (5551.5–6879.0) | 3800.4 (1318.9–8002.4) | 8.2 (1.1–11.7)* | 2367.6 (1617.5–2929.5) | 2371.5 (1596.4–3007.5) |
| MIP-1α | 24.86 | 36.4 (22.8–46.6)* | 8.5 (7.1–9.6)* | 128.5 (106.3–151.2) | 111.0 (52.7–173.7) | 6.9 (6.2–8.0)* | 41.4 (33.8–48.4) | 125.3 (79.7–171.5) |
| MIP-1β | 11.0 | 40.6 (20.2–57.9) | 0.6 (BLD, 0.6)* | 270.3 (232.9–328.9) | 621.4 (146.8–1337.8) | BLD | 95.8 (68.3–148.8) | 490.3 (150.8–827.6) |
| MIG | 5.19 | 9.6 ^‡^ | 27.1 (0.7–52.6)* | 479.5 (173.1–712.7) | 42.9 (18.8–59.3) | 22.3 (5.2 – 33.3)* | 112.1 (45.3–160.3) | 64.2 (12.6–154.6) |
| GM-CSF | 6.86 | 1311.9 (1095.0–1560.0) | 2890.6 (2530.2–3335.0) | 3191.4 (2035.3–4209.1) | 2002.9 (1497.9–2328.2) | 3609.4 (2652.1 – 4051.5) | 2261.5 (1730.0–2934.8) | 2714.4 (1651.5–4283.5) |
| RANTES | 16.21 | 25.4 (21.7–29.6) | 3.7 (1.8–5.7)* | 6.6 (4.4–8.7)* | 12.9 (6.7–21.8)* | 2.4 (0.0 – 4.3)* | 8.2 (4.1–11.5)* | 38.6 (24.4–52.8)* |

*, one or more values within group below the limit of detection or outside of the dynamic range; ‡, other values in group indeterminant or BLD; BLD, below the limit of detection; LLDR, lower limit of dynamic range; Lo, inoculation with 10 TCID_50_ of RVFV; Hi, inoculation with 10^4^ TCID_50_ of RVFV; NSGS, unengrafted NSG-SGM3 mice; 13-wk, humanized mice inoculated at 13 weeks post engraftment; 19-wk, humanized mice inoculated at 19 weeks post engraftment.
