# Supplementary material for: Human immune cell engraftment does not alter development of severe acute Rift Valley fever in mice
Source: PLoS One. 2018 Jul 20;13(7):e0201104. doi: 10.1371/journal.pone.0201104 (PMC6054394; doi:10.1371/journal.pone.0201104)
Supplement: S2 Table — Mouse cytokine levels in plasma of mock- and RVFV-inoculated humanized mice at terminal timepoints. Historical samples from mock-inoculated (control) SGM3 humanized mice were used to determine baseline expression. Values are expressed as the mean (range) in pg/mL. (DOCX) [file pone.0201104.s002.docx]

**S2 Table. Mouse cytokine expression in plasma of mock- and RVFV-infected humanized mice.** Mouse cytokine levels in plasma of mock- and RVFV-inoculated humanized mice at terminal timepoints. Historical samples from mock-inoculated (control) SGM3 humanized mice were used to determine baseline expression. Values are expressed as the mean (range) in pg/mL.

|  |  |  | **RVFV-inoculated** | | | | | |
| --- | --- | --- | --- | --- | --- | --- | --- | --- |
| **Cytokine** | **LLDR (pg/mL)** | **Control** | **Hi-NSGS** | **Hi 13-wk** | **Hi 19-wk** | **Lo-NSGS** | **Lo 13-wk** | **Lo 19-wk** |
| IL-1β | 1.00 | BLD | 18.8 (16.6–20.2) | 10.7 (5.6–20.3) | 7.2 (5.1, 9.3) | 12.1 (5.5–18.7) | 3.2 (2.9, 3.5) | 10.8 (3.0–19.2) |
| IL-2 | 1.20 | BLD | 10.9 (8.8–12.0) | 3.9 (0.2–9.9)* | 4.6^‡^ | 8.3 (2.0–12.8) | BLD | 7.4 (3.1–11.8)* |
| IL-4 | 1.27 | 2.5 (1.9–3.0) | 13.3 (12.0–14.4) | 10.1 (7.9–13.0) | 8.6 (8.5, 8.7) | 11.7 (9.2–13.7) | 6.7 (6.5, 7.0) | 9.6 (7.7–12.3) |
| IL-5 | 2.13 | 0.8 (0.5–1.1)* | 40.8 (37.7–44.6) | 31.2 (24.5–42.6) | 23.8 (21.5, 26.1) | 31.0 (19.9–42.1) | 19.4 (17.8, 21.0) | 28.1 (16.2–41.0) |
| IL-6 | 4.10 | 4.1^‡^ | 23077.9 (11307.8– 34092.0) | 6874.5 (1280.8–15466.8) | 2982.8 (2794.1, 3171.5) | 10412.0 (1649.0– 27467.5) | 668.4 (290.5, 1046.2) | 11166.4 (1195.4–23120.2) |
| IL-9 | 13.82 | 34.8^‡^ | 266.3 (222.0–296.8) | 216.5 (101.8–400.8) | 157.6 (133.0, 182.1) | 234.2 (157.8–290.3) | 117.1 (90.0, 144.1) | 186.1 (90.0–298.1) |
| IL-10 | 2.02 | BLD | 621.8 (347.7–739.7) | 346.0 87.3–715.0) | 255.1 (169.2, 341.0) | 454.2 (167.1–720.9) | 67.9 (50.4, 85.3) | 351.8 (56.5–732.7) |
| IL-12p70 | 3.62 | 1.4 (0.9–1.9)* | 71.2 (61.6–77.6) | 36.5 (12.8–67.9) | 33.8 (22.1, 45.4) | 46.9 (17.6–73.6) | 11.4 (9.9, 12.8) | 43.4 (12.6–74.5) |
| IL-13 | 2.37 | 2.7^‡^ | 14.9 (10.0–17.2) | 7.9 (1.6–15.9)* | 6.6 (4.1, 9.1) | 11.3 (4.0–17.1) | 22.5 (1.6, 43.3) | 9.9 (3.9–16.9) |
| IL-17A | 1.12 | BLD | 43.6 (38.8–46.5) | 21.0 (6.8–41.1) | 18.5 (14.8, 22.2) | 26.3 (10.5–39.6) | 6.5 (5.1, 8.0) | 31.4 (24.3–40.5) |
| IL-18 | 29.57 | 296.7 (114.0–625.6) | 10930.8 (6722.4–13209.5) | 10438.0 (5011.0– 17942.6) | 8836.8 (7749.3, 9924.2) | 9719.6 (6486.7–12844.7) | 6105.3 (5901.6, 6309.0) | 10484.8 (2713.0–20925.5) |
| IL-22 | 10.81 | BLD | 136.1 (113.6–158.5) | 65.8 (12.2–136.1) | 57.7 (48.0, 67.4) | 84.1 (30.2–125.4) | 14.9 (12.2, 17.6) | 72.1 (20.2–124.3) |
| IL-23 | 10.95 | BLD | 234.6 (190.3–253.8) | 105.7 (15.8–226.9) | 88.0 (57.0, 119.1) | 165.5 (74.5–245.5) | 20.9 (18.1, 23.7) | 121.8 (26.9–213.6) |
| IL-27 | 2.31 | BLD | 36.4 (32.1–41.9) | 56.3 (12.8–136.8) | 20.9 (14.4, 27.3) | 27.1 (15.2–36.2) | 20.8 (9.5, 32.1) | 23.5 (7.9–40.3) |
| IP-10 | 0.37 | 15.0 (10.7–18.7) | 402.0 (174.5–543.6) | 301.9 (192.1–399.8) | 228.6 (224.5, 232.8) | 318.6 (272.4–354.4) | 245.3 (225.5, 265.1) | 347.8 (337.1, 357.0) |
| IFN-ɣ | 0.94 | 0.0^‡^ | 16.9 (15.4–17.8) | 8.2 (2.6–16.4) | 6.4 (4.5, 8.2) | 10.4 (3.9–16.6) | 1.9 (1.7, 2.2) | 8.4 (1.9–15.5) |
| TNF-α | 0.89 | 0.3^‡^ | 60.6 (56.1–66.0) | 45.9 (17.1–75.3) | 36.7 (28.6, 44.8) | 39.5 (15.0–63.7) | 16.3 (13.0, 19.5) | 56.5 (19.1–93.6) |
| Eotaxin | 0.50 | 854.3 (665.1–1176.9) | 3177.9 (2601.6–3754.3)^‡^ | 2947.8 (2123.7– 3534.1) | 2162.8 2011.2, 2314.4) | 2400.0^‡^ | 2649.0 (2367.6, 2930.4) | 2098.9 (1921.2–2338.2) |
| GRO-α/KC | 1.49 | 26.2 (17.5–34.9) | 1365.7 (942.9–1877.0) | 6888.1 (1872.4– 16381.5) | 4808.9 (3955.0, 5662.9) | 877.3 (367.1–1219.5) | 1280.7 (865.4, 1696.0) | 4250.9 (493.3–9463.4) |
| MCP-1 | 5.80 | 91.2 (79.6–108.8) | 37395.8 (18233.7–66088.4) | 15383.2 (12619.9– 18373.8) | 13844.9 (13108.3, 14581.6) | 17402.4 (9247.7–31411.4) | 10605.1 (8272.9, 12937.3) | 17120.0 (12033.1–216690.6) |
| MCP-3 | 0.23 | 287.5 (179.0–442.2) | 1106.7 (987.3–1226.2)^‡^ | 1087.5 (767.8–1277.7) | 904.4 (866.6, 942.3) | 1092.3 (1042.3–1128.7) ^*^ | 956.3 (913.3, 999.3) | 1016.4 (889.1–1177.2) |
| MIP-1α | 0.41 | BLD | 8.0 (5.6–11.5) | 8.3 (7.3–9.7) | 8.4 (7.5, 9.2) | 6.6 (4.4–8.8) | 6.8 (5.2, 8.5) | 12.3 (9.0–16.8) |
| MIP-1β | 1.03 | 1.3 (1.0–1.6)* | 44.4 (28.8–50.9) | 20.6 (16.0–29.7) | 26.9 (22.1, 31.6) | 24.2 (16.0–34.3) | 18.2 (14.1, 22.3) | 54.6 (32.2–84.8) |
| MIP-2 | 0.54 | 5.6 (3.3–9.7) | 832.0 (528.3–1396.5) | 747.6 496.6–1196.4) | 987.9 (713.2, 1262.7) | 525.1 (300.4–798.7) | 596.3 (329.5, 863.0) | 1263.1 (67.4–2678.5) |
| GM-CSF | 1.82 | BLD | 79.0 (73.5–84.5) | 45.8 (20.6–75.7) | 42.7 (32.1, 53.3) | 54.8 (27.9–77.3) | 17.7 (16.4, 19.0) | 48.8 (17.5–76.6) |
| RANTES | 2.47 | 3.8 (0.8–6.7)* | 225.0 (141.8–272.2) | 282.0 (218.4–350.2) | 173.8 (136.3, 211.3) | 216.8 (192.3–230.5) | 162.3 (159.7, 164.9) | 157.1 (100.2–207.2) |

*, one or more values within group below the lower limit of the dynamic range. ‡, other values in group indeterminant or BLD; BLD, below limit of detection; LLDR, lower limit of the dynamic range; Lo, inoculation with 10 TCID_50_ of RVFV; Hi, inoculation with 10^4^ TCID_50_ of RVFV; NSGS, unengrafted NSG-SGM3 mice; 13-wk, humanized mice inoculated at 13 weeks post engraftment; 19-wk, humanized mice inoculated at 19 weeks post engraftment.
